# Building community resilience during COVID‐19: Learning from rural Bangladesh

**DOI:** 10.1111/1468-5973.12405

**Published:** 2022-03-27

**Authors:** Farooq Ahmad, Rashedur Chowdhury, Benjamin Siedler, Wilson Odek

**Affiliations:** ^1^ Southampton Business School University of Southampton Southampton United Kingdom; ^2^ Michael Smurfit Business School University College Dublin Dublin Carysfort Avenue, Blackrock Ireland

**Keywords:** community resilience, Covid‐19, crisis, lockdown, marginalized community

## Abstract

The COVID‐19 pandemic has brought overwhelming challenges to developing countries which are already resource‐constrained and lack adequate social safety nets. Specifically, lockdown has adversely impacted marginalized communities (e.g., labourer, fish wholesaler and small business owner) and informal sector employees who rely on meager daily wages for their survival. Set in the contested climate of the emergency response to the COVID‐19 outbreak in Phulbari, Dinajpur, Bangladesh, we examine the early response of the community to the pandemic. Drawing on 24 in‐depth interviews with members of this community, we find that the existing central and regional government structure has failed to deal with the crisis. Yet, we show how collective effort at the local community level, led by volunteers and community leaders, is crucial in the fight against hardship during lockdown.

## 1 INTRODUCTION

In the current COVID‐19 pandemic, shelter‐in‐place and social distancing policies have been implemented around the world to stop the spread of the virus. However, in developing countries, the effectiveness of stringent confinement policies is strongly questioned due to the disastrous socioeconomic consequences (Arndt et al., 2020). Rashid et al. (2020, p. 2) point out that the idea of lockdown ‘has been imported from western or developed countries with stronger economic bases and better social safety nets for those in need’. Policymakers need to recognize the severe socioeconomic inequalities that exist in developing countries and the disastrous consequences of preventing thousands of people from working. The reality in developing countries is that a large part of the population works in the informal sector. This means that a huge number of people rely on daily income from hands‐on labour, which is impossible to continue under strict self‐isolated restrictions (Robalino, 2020). Without adequate financial aid, the poor in developing countries cannot obey lockdown rules because they need to feed themselves and their families (Gerard et al., 2020).

Recent research (e.g., Arndt et al., 2020; Buheji et al., 2020; Chirisa et al., 2020) and media reports (e.g., Naqvi, 2020; Shahid, 2020; Siddiqui, 2020) on the early lockdowns indicate that they render marginalized communities very vulnerable to hunger. These studies further suggest that poor management could lead to a starvation crisis with a far worse impact than that of the virus (Mishra & Rampal, 2020). For example, Rashid et al. (2020) show the devastating effect of the lockdown on slum‐dwellers in Bangladesh who have no access to food unless they can work. Consequently, whole communities are navigating their way through strong negative emotions: fear, anger, depression, hopelessness, and despair. In India, thousands of casual workers took to the streets in protest because they were unable to feed their families. Although it may be stating the obvious, self‐isolation and social distancing are the last things on the minds of a starving population. Emerging studies show that community resilience has long been and continues to be a viable strategy, as people search for ways to deal with and navigate the effects of COVID‐19 policies (e.g., Fransen et al., 2021; South et al., 2020). A recent study by Abdalla et al. (2021), however, indicates that during the COVID‐19 pandemic local communities also demonstrated positive feelings (e.g., empathy and solidarity), attitudes (e.g., social responsibility), and behaviours (e.g., mutual aid and cooperation), which led to community resilience emerging in many localities across the world.

Academic discourse (Imperiale & Vanclay, 2016, 2021; Norris et al., 2008) and United Nations declarations (UNDP, 2014; UNISDR, 2015) suggest that community resilience plays an important role in mitigating the effects of the disaster because it empowers the local community to help the vulnerable. Community resilience is defined as the activation of socially motivated processes by local people who are committed to working together to prevail against adversity to keep their community alive and well (Imperiale & Vanclay, 2021). However, to date, the academic debate on the role of community resilience is based mainly on disasters caused by natural events and accidents such as floods (Aijazi & Panjwani, 2015; Ntontis et al., 2020; Qasim et al., 2016; Van De Lindt et al., 2020; Wickes et al., 2017), earthquakes (Imperiale & Vanclay, 2016, 2020a; Thornley et al., 2015), tornados (Houston et al., 2017; Spialek et al., 2016; Wang et al., 2018) and chemical spills (Reams et al., 2017), while there is a paucity of research focusing on a pandemic situation as a disaster event framework. The restricted mobility and interaction that is a unique consequence of pandemic disasters as opposed to natural hazard‐related disasters may limit the ability to leverage social capital to respond to hazard.

Unlike our current knowledge of naturally occurring disasters, we know very little about community response to human‐induced disasters such as health‐related catastrophes (Erikson, 1994; Rao & Greve, 2018). Therefore, we aim to investigate how community resilience is an effective strategy to deal with the catastrophe caused by the pandemic within the context of government policy proving to be more of a threat to the poor and vulnerable than the disease itself is. Engaging with the field‐level experiences of early responders can yield valuable insights into the under‐examined topic of community resilience in disaster environments created by a pandemic. Considering a community in Phulbari, Dinajpur, Bangladesh, as a case study, our research examines the development of local community resilience during the first COVID‐19 lockdown, beginning in March 2020. Our findings indicate that in countries with inadequate disaster management plans and limited government capacity, the role of community resilience becomes ever more crucial to tackle a humanitarian crisis in the making, particularly in the crisis created by the pandemic outbreak.

## 2 DISASTER MANAGEMENT THROUGH COMMUNITY RESILIENCE

Academic research on disaster management (e.g., Aldrich, 2017; Chandra et al., 2013; Imperiale & Vanclay, 2016, 2021; Norris et al., 2008), as well as United Nations reports (e.g., UNDP, 2014; UNISDR, 2007, 2015), indicate that community resilience is an essential component of disaster management and can prevent the onset of crisis. Specifically, community resilience is now recognized as an important contributory factor in the field of public emergency management, which ‘emphasizes the assessment of community strengths and not simply the description of vulnerabilities’ (Plough et al., 2013, p. 1191). The notion of community resilience is increasingly being applied across a range of public policy, planning and management discourses as a means of addressing the uneven ability of local communities to respond to changes wrought by social, economic, and political processes (Chandra et al., 2013).

Chandra et al. (2013) state that the five core components of community resilience are (i) physical and psychological health, (ii) social and economic equity and wellbeing, (iii) effective risk communication, (iv) integration of governmental and nongovernmental organizations, and (v) social connectedness. Norris et al. (2008) describe community resilience as a set of networked adaptive capacities, including economic development, information and communication, community competencies, and social capital. Very recently, Imperiale and Vanclay (2021) offer a more nuanced understanding of it as comprising a number of different social processes or dimensions. These include cognitive processes such as feelings, attitudes, needs, desires, beliefs, and values as well as the interactional dimension that includes issues such as mutual aid, social equity, inclusion, community wellbeing, and awareness of risk and sustainability that are actioned by the collective efforts of local people. Unexpected change such as crises, disasters and other social disturbances force local communities to pull together for their own survival and, in the process, they learn how to maximize local capabilities, mitigate risks and impacts, and combat the threat to community wellbeing at all levels (Cavaye & Ross, 2019; Imperiale & Vanclay, 2016; Matarrita‐Cascante et al., 2017).

Research on community resilience indicates that communities that are well‐trained socially and psychologically are more likely to respond, adapt and bounce back quickly from catastrophic events (Aldrich, 2017; Mathbor, 2007; Norris et al., 2008; Patterson et al., 2010; Putnam, 2000). Studies in this area further show that resilient communities are able to identify the causes of crisis and make changes to their structure and functioning to adapt, transform, and survive (Aldrich & Meyer, 2015; Mayer, 2019; Plough et al., 2013). At its best, community resilience addresses the plight of all the vulnerable sectors of that society and devises measures to prepare for, avert, and mitigate future crises. This occurs through specific social processes of social learning and transformation (Imperiale & Vanclay, 2021) in which communities learn from their previous experiences and move towards sustainability.

Research on disasters suggests that community resilience is particularly vital in developing countries, given their fragile institutional structures and limited capability to support their vulnerable populations (Aldrich, 2017, Jacobson et al., 2018, Rashid et al., 2020). In this regard, disaster research is at the forefront of understanding how community resilience develops in the face of disturbance, stress, or adversity (Cutter et al., 2008, Imperiale & Vanclay, 2021). Studies show that community strategies based on equity and social justice can help substantially in disaster damage limitation and the rebuilding of lives and livelihoods (Plough et al., 2013). To date, however, research on disaster and community resilience is subject to at least two limitations.

*First*, existing research tends to focus on disasters associated with natural hazards such as earthquakes, tornadoes, chemical spills, fires, and floods, and there is a paucity of research that focuses on the disaster caused by the ‘pandemic outbreak’ and human intervention. Research in this area (e.g., Cavaye & Ross, 2019; Imperiale & Vanclay, 2016, 2021; Matarrita‐Cascante et al., 2017) also posits that resilient societies learn from previous experiences of crisis through the social processes of ‘social learning’ and ‘transformation towards sustainability’; thus, they cope better in the future. However, it is important to acknowledge that—given the different dynamics of natural hazard‐related disasters, such as restricted mobility and interaction limiting the ability to leverage social capital to respond to hazard events, and the pandemic as a disaster framework—we know very little about community response to such catastrophes (Erikson, 1994, Rao & Greve, 2018).

*Second*, although a number of attempts have been made to explore how disaster victims recover through community resilience, the effects of economic and social inequities experienced by marginalized communities in such situations have rarely been addressed in the disaster and community resilience literature (Walters, 2015). Thus, this study aims to address this limitation by examining how a marginalized community responds to the ongoing pandemic and how this disaster is impacting vulnerable groups beset by social inequalities.

## 3 METHODOLOGY

### 3.1 Case study: The impact of a lockdown in the community of Phulbari

Our study is based in a rural marginalized community in Bangladesh. Bangladesh is situated in South Asia and has a population of approximately 164.7 million (Rahman et al., 2021). The World Bank (2020) estimates that almost 63% of Bangladeshis reside in the rural areas. The country has a devolved administrative structure with eight divisions, 64 districts (known as Zila), 492 subdistricts (known as Upazilas) and 4554 union parishads (Salehin et al., 2020).

We selected the Phulbari subdistrict from the list of 492 subdistricts due to the years of networking and in‐country research experience of one of our lead researchers. Phulbari is a unique case study in that, despite being marginalized, they have a history of community resilience having vehemently fought off an exploitative open pit coal mining scheme that threatened the lives and livelihoods of the locals (Chowdhury, 2021). Thus, studying their community resilience in the context of a pandemic will help us gain valuable insights into just how the community manages to resist life‐threatening instances and prevails.

**Figure 1:**
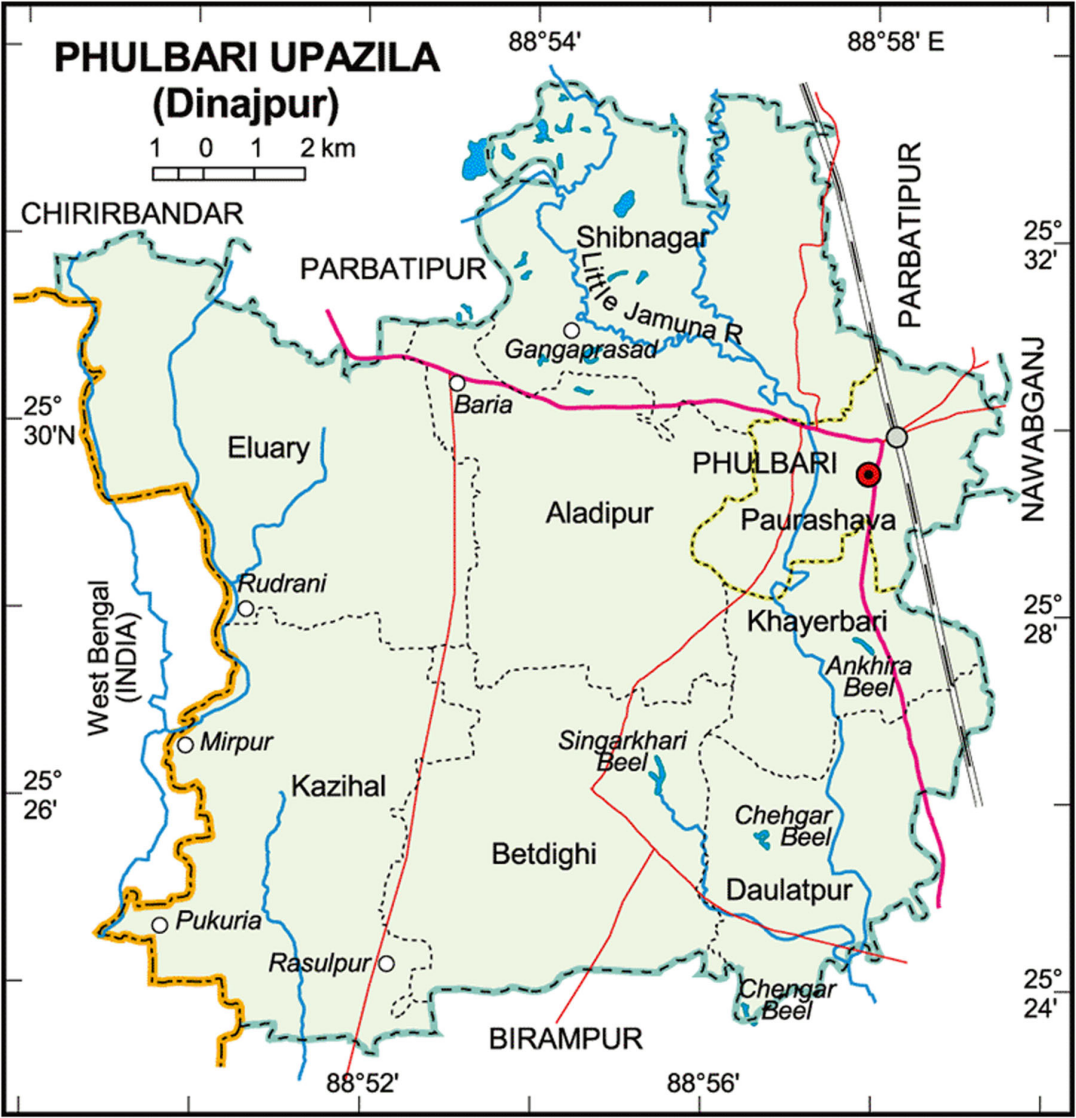
Map of Phulbari Upazila and surrounding areas. *Source*: Bangladesh National Portal (2021)

Phulbari subdistrict is found in the Dinajpur district in the Division of Rangpur, Bangladesh. As at their last census in 2011, the subdistrict has a population of 176,023. The average household size in the region is just above four, with a literacy level of around 28% (Bangladesh Bureau of Statistics, 2015) (Figure 1).

At the time of writing, the COVID‐19 pandemic had not raged through Phulbari, as it had done in many other cities, or through wider Bangladesh, where over 1.5 million cases of COVID‐19 and nearly 28,000 deaths had been reported (Dhaka Tribune, 2021). However, when the interviews were conducted, active cases of COVID‐19 had been reported in the nearby subdistricts of Phulbari (Ghosh & Mollah, 2020). Thus, government regulations on social distancing and increased hygiene to prevent further outbreak were being implemented. COVID‐19 containment and preventive measures have been largely national government‐driven, with minimal resource availability to drive expansive efforts.

There remains a low level of testing capacity, given the population size and difficult outreach of all members of society (Rahaman et al., 2020), which also hinders effective containment measures. More detailed descriptions of localized interventions and recovery efforts carried out by local leaders drawn from the experiences of our interviewees are laid out below.

### 3.2 Data sources and analysis

This study is rooted in the phenomenological tradition of qualitative research that seeks to make sense of or interpret phenomena in terms of the meanings that people confer to them (Denzin & Lincoln, 2011). Hence, to understand how vulnerable communities were impacted by the lockdown and how they responded to the crisis, we relied on interviews with community members. These included low‐income casual workers comprising one construction worker, one fruit seller, two fish wholesaler, and two shopkeepers who depended on regular daily wages for survival but who were forbidden to trade during lockdown. We also managed to secure interviews with two local politicians who have grassroots knowledge of their communities, alongside two representatives from local NGOs, one madrasa teacher, two imams, two schoolteachers, two small business owners, two union leaders, one journalist, one nurse, and three community members and leaders who had experience of helping the vulnerable during the pandemic. The interviews, which were conducted in Bengali, lasted between 60 and 90 min. All the interviews were tape‐recorded, and informed consent was obtained for each interview.

Our interviews took place in June 2020, during the first wave of the COVID‐19 pandemic, which gave a real‐time perspective of the catastrophe as it was unfolding.

In sum, we conducted 24 in‐depth interviews (with the help of five research assistants) in Phulbari with a representative sample of the community who had responded in some way to the COVID‐19 lockdown, owing to their social position or formal affiliation. We used a combination of purposive and snowball sampling techniques. In the purposive sampling we selected participants according to three criteria:

- Participants had to belong to a marginalized community that was severely impacted economically by the lockdown,
- Participants had to have been involved and engaged in rescuing or helping vulnerable members of the community,
- Participants had to be representative for their local community.

We used a snowballing technique—that is, we recruited participants based on recommendations from existing interviewees with insider knowledge of the ground‐level rescue process. For example, two imams and a school teacher were recruited based on the recommendation of informants who had already been interviewed and had talked about other members of the community who were actively engaged in fundraising for the poor.

The respondents at the community level (casual worker, construction worker, transport leader, fruit seller, fish wholesaler, businessman, NGO representative, school teacher, shopkeeper, nurse, imam, madrasa teacher, journalist, union leader) were asked how the pandemic and the lockdown had affected their community. Topics included the initial local community response, the role of each community member, the effectiveness of different community roles, local and central government responses and interventions, the role of NGOs and civil society, and disaster preparation for the community for the future. Respondents at the political level (local politicians, the mayor of Phulbari, and the union parishad chairperson) were asked additional questions about local and central government interventions and the existing emergency response program. Although we used semi‐structured questionnaires, all participants were encouraged to tell the stories they thought were most important regarding community response to and recovery from the COVID‐19 crisis.

Three research assistants helped us to translate all the interviews. Following Braun and Clarke (2006), the co‐authors conducted a thematic analysis of the interview transcripts to identify the common themes emerging within the data. First, a set of descriptive codes was generated, followed by analytical coding, which together revealed the explicit and implicit data of participants’ experiences. Second, we identified specific themes with reference to the literature. Specifically, through coding and evaluation of the interview data against the theoretical background identified in the literature review, we were able to inductively develop our theoretical themes. Finally, we arranged and organized the themes in relation to verbatim data extracts.

## 4 THE DRIVERS OF COMMUNITY RESILIENCE IN PHULBARI DURING THE COVID‐19 OUTBREAK

In the first weeks of the outbreak, community members self‐organized and helped each other. This strategy of community resilience proved vital to the successful management of this unique catastrophe. Based on observations and discussions with interview respondents, we identified the following initiatives taken by community members.

- Resilience‐building through improvisation of local government structure
- Resilience‐building through neighbourhood action
- Resilience‐building through community leadership
- Resilience‐building through recruiting young volunteers

### 4.1 Resilience building through improvisation of local government structure

Our analysis reveals that the existing centralized government structure tends to hinder the prompt action necessary to help the vulnerable. Study informants pointed out two ways in which central and local government bodies could adapt their functionalities to provide better aid and facilities to the poor. Our informants at the community leadership level suggested that there should be much greater coordination between central and local governments so that resources could be more quickly generated and fast‐tracked to the needy. They were keen that an intergovernmental relationship should minimize bureaucratic processes since time is of the essence in a disaster scenario. One local politician was concerned about how much the vulnerable were suffering:

The government is saying that they are giving enough aid, but in reality, we have to wait as it comes from central government to the local authorities and only then to the areas that need it. I think there is need of a swift mechanism between central and local government for the fast delivery of resources.

The respondents felt that local governments on their own were unable to deal with the catastrophe due to limited resources and expertise, and that support from central government was an absolute requirement. They criticized the existing command and control structure in which local government officials are forced to wait for orders to be issued by the central government. This form of top‐down governmentality results in complex bureaucratic procedures which, in the current context, delayed the aid and recovery processes. Subsequently, this created anger and mistrust against central and local governmental organizations. One of the respondents, a transport union leader, highlighted the importance of intragovernmental relationships for prompt resource mobilization:

There is urgency to establish a relationship between central and local governments so that communities can receive financial and logistical help more quickly, like help from the army. The army could deliver aid straight to people's houses.’

He further added:

The army could secure this lockdown better than police administration because people don't trust the police that much.

One of the local politicians suggested that, to help the vulnerable more effectively,

…the Minister of Health should establish an emergency core committee led by disaster management experts. However, it is highly important that each district should be represented by a core group of local leaders, local government officials, and expert doctors. The local leadership can disseminate grassroots information and outline the financial aid they require. Throughout this process the intragovernmental coordination mechanism should be free of red‐tape and bureaucracy during the crisis.

A second suggestion concerns political empowerment at the local level. Those of our study informants who were local leaders observed that the current government's centralization of power has created barriers to helping the vulnerable in a timely manner. Centralization tends to widen the gap between government planning at the national level and implementation within communities at the local level. Since most of the poorer population live at a distance from central administration, they depend on local government to deliver aid and guidance. A union parishad chairperson described the problem:

We have limited funds and resources and so the central government has to send emergency funds. We are trying our best in our constituencies, but we are up against financial limitations.

Similarly, according to a local politician,

We have created a fund through an initiative taken by the local political party. We have tried to use this to help the poor and vulnerable.

Another local leader commented on local authorities working in the community:

We consider the steps that are being taken by the local government, including the UNO, to be very important and we are following them to the best of our ability. He (divisional commissioner) visits the area in person to investigate conditions as well as to monitor whether we are following his instructions to the letter. I'm grateful to him because he's taken useful steps to keep us safe from coronavirus.

The respondents are clearly in favour of local government empowerment, since leaders at the grassroots level have a much stronger knowledge of the communities they serve and work in under the auspices of central government to implement relevant strategies in urban and rural areas. Consider the following quotation from a union parishad chairperson:

I've been elected by this constituency many times; these are my voters… I have more knowledge about my people than a minister in central government.

Further, the respondents stressed the importance of collaboration with local opposition parties and the need to abandon power politics in times of crisis in favour of working together to help the marginalized. Such an approach has resonated with the community, particularly in instances where political figures have gone beyond the call of duty to initiate ventures for the continuity or resumption of normalcy as far as is possible. The Mayor added,

The government is hinting at collaboration from all political parties. The government is bringing together the UNOs and other administrative officers from all of the 64 districts. These are people who are in direct contact with the community.

In sum, the central government needs to adapt to be able to respond rapidly to the needs of the most vulnerable. Strong relationships between central government and local authorities are needed to facilitate the timely distribution of resources. Informants at the leadership level stressed the need for better coordination between all government agencies and the formation of cross‐functional teams to manage the pandemic response more effectively. Respondents also emphasized the need for both central and local governments to fast‐track collaboration to distribute resources for basic social needs quickly and efficiently. In addition, they felt that central government should confer more powers to community leaders to enable a swift and more effective response. Local government currently lacks the necessary resources as, financially, it is fully dependent on central government, and there is a call from community leaders for a decentralized form of responsibility where they address the interests and survival of all their electorates.

### 4.2 Building resilience through neighbourhood: Encouraging and supporting local community action

Our analysis reveals that the neighbourhood as a local community was at the forefront of community resilience. In this context, ‘community’ refers to social subsystems including families, neighbourhoods, villages, cities and divisions. In the context of our study, ‘local community’ refers to families, friends and residents living in the same neighbourhood. Most of the respondents called on the local people for support to help the marginalized in their own capacity. An imam of a local mosque explained:

All our neighbors in the village; the youth community, the teachers, those who are doing business, all of us together have raised money and distributed rice, lentils, potatoes, soap etc. to the poor and helpless. This is the only way to help society.

He further added that the community should shoulder greater responsibility for the problem and help those who are severely impacted by the lockdown:

The local community shouldn't rely only on government planning. This is our village; we have to be conscious about this on our own.

This demonstrates his inherent sense that external aid is unpredictable, and that the community needs to raise awareness and generate internal support systems. Another local teacher emphasized the collective efforts of the community:

Collectively we will all try to prevent the situation from becoming worse. We have two community associations, and we can all try and see what we can do.

Similarly, a doctor who works in the local hospital expressed his desire to help:

Those of us who are solvent, we can do something, we can arrange food for them. If we can provide for them, they will be able to stay at home for a month. If we can arrange that, then there would be far fewer problems.

A local politician explained how affluent locals are helping the vulnerable:

We are trying to help people by providing social aid. The more well‐off people in the area are gathering together to raise money to distribute food to those of our community who are in need.

Similarly, according to a union parishad chairperson:

I would like to invite the more affluent in our neighborhoods to extend a helping hand to the people experiencing poverty in this area.

Several interviewees echoed the view that local communities need to come together in support of those who had lost their jobs as a result of lockdown. The willingness to share responsibility and comply with community decisions could be utilized to harness resilience.

### 4.3 Building resilience through community leadership

Several respondents in our study pointed out the importance of community leadership in the current lockdown situation. Our definition of leadership here is broad and includes local politicians, community elders and religious leaders who exercise great influence over local communities. Our study informants indicated that the role of local community leaders was crucial at this time as they have a deeper knowledge of the day‐to‐day lives of their communities and respond promptly to the needs of the vulnerable. Consider a quote from a local shopkeeper who discusses the importance of a local leadership:

Our local Nazim is very active in his constituency—he attends the marriages and funerals of his constituents, and he knows where the slums are and where the vulnerable in his community live.

In the Bangladeshi context, community elders have a great influence over the local community and are held in high esteem. Our respondents indicated that their community elders had raised considerable amounts of money to aid the vulnerable by visiting individual households and asking for donations. The community elders have engaged in successful fundraising not only to feed the vulnerable but also to educate the community about adhering to guidelines to avoid the spread of the virus. These sentiments are illustrated by a fish wholesaler in the following quotation:

The fundraising efforts of the community elders are praiseworthy. They are always trying really hard and constantly instructing us to maintain social distancing. They hand out educational leaflets as well.

Similarly, a school teacher noted,

They are working verbally and giving advice to the people of this area.

The respondents also mentioned the positive role of imams, who they were grateful to see inspiring the community to help the vulnerable and build resilience. Bangladesh is a largely Islamic country where religious leaders and imams are well regarded. They are strongly connected to their followers and, thus, their advice can greatly shape the day‐to‐day decisions of communities. Imams are reminding their communities that charitable donation is a religious obligation that ranks second only to prayer. These existing channels, as one imam says, are enshrined in the Holy Quran:

The rich are obliged to distribute a portion of their earnings among the poor, as zakat.

Similarly, according to another respondent who is an imam in the local mosque:

Yes, I have given them advice; the people who are poor have a right to the wealth of the solvent.

Our analysis revealed that while imams were aware of the basic principles of social distancing and handwashing, they were also keen to interpret the pandemic as a punishment from God. An imam at a local mosque stated,

I mentioned earlier that this is a punishment from Allah (SWT). What they have already said, I totally agree with, there is nothing to disagree upon. However, it would be better if this came from Allah and we don't have any other option but to surrender to Allah. The things that made us ashraful maqlukat, the best creature, are not within us anymore. Beastly nature lives within us now. If we do this, then we and the animals will be differentiated. We have to ask for forgiveness from Allah. Whatever the doctors and scientists are telling people is all good, but we have to ask for forgiveness from Allah as well. Did we ever see, hear or read about something like this? So, we have to ask for forgiveness from Allah (SWT).

According to another local imam:

There is not much to discuss but we must do good work to save ourselves from the punishment of Allah. We have to pray, we have to remain on the path of honesty, the rich have to distribute portions of their earnings among the poor.

Although imams view the pandemic as a punishment from God and their views on women and adultery are highly problematic and concerning, they are also advising their congregations to comply with government guidance on combating the spread of the virus. An imam claims how they have been following governmental advice in the mosque:

Between five and seven people, including the imam and muezzin, can pray inside the mosque. We gave orders for everyone to pray from their homes. That's it. We have instructed them to make as few visits to the markets as possible, although it would be better if they didn't go at all. If you have to buy something for your household, there should be only one person going out to get it.

Since imams are influential within their communities, they play a pivotal role in how the pandemic is managed. The different types of local leader (e.g., politicians, community elders and clerics) also contribute to community resilience by motivating their followers to give charitably to the vulnerable in whatever way they can. Leaders, as part of the community, can help to raise awareness of the plight of low‐income workers and others who are suffering financially in the current crisis. By educating the public there is a greater likelihood of communities pulling together to observe government guidelines and helping each other to access basic living necessities.

### 4.4 Building resilience through engaging young volunteers

Our analysis reveals that another driver of community resilience was young volunteers. In the beginning the behaviour of younger people was misaligned with community efforts; however, with the combined efforts of schoolteachers and the advice of community elders, the volunteers subsequently became more engaged in community activities and ultimately proved to be important drivers of community resilience in the neighbourhoods. The noncompliance of the younger generation was noted by local religious and administrative leaders who then began lobbying them. The mayor was proud to say:

I recruited teams and committees from among the younger people in our community. They are working to maintain social distancing and are making sure that people from outside don't come into our area.

This involvement and engagement encouraged young people to take even more responsibility for their community. The results were immediately noted with appreciation by one of the local nurses who stated that:

The local young population are working together to help the community maintain good hygiene and safety measures. They are also good at raising awareness, distributing food and supplies, and even helping the poor financially.

However, one of the local doctors felt that the volunteer scheme could have been better organized:

We didn't discuss the distribution of volunteers and so some areas are without any help. It would have been better if they had been assigned to work in all areas, because then the initiatives to maintain social distancing would have worked better.

In sum, engaging younger volunteers to help in the community has ensured goal congruence as the lockdown continues. As volunteers participate in delivering food to the poor, they simultaneously work to make the community aware of why social distancing is important.

## 5 DISCUSSION

The COVID‐19 lockdown drastically reduced the incomes of many workers and others living on the breadline in developing countries, creating ever wider social inequalities and poverty. Because of this, the role of community resilience in feeding, clothing and sheltering the most vulnerable is perhaps more vital than ever before. The field of community resilience research builds on strong evidence of community cohesion, the influence of neighbourhoods on individual and community wellbeing, social equity, trust and knowledge acquisition and transfer (Aldrich, 2017, Almutairi et al., 2020, Imperiale & Vanclay, 2016; Norris et al., 2008; Pfefferbaum et al., 2017). We have sought here to advance our understanding of community resilience in a pandemic situation, using the case of the COVID‐19 lockdown in Phulbari, Dinajpur, Bangladesh.

The existing disaster relief literature fails to sufficiently highlight the tensions between impacted communities and the relief process. The current focus of related research explores the dynamics of aid effectiveness including analysis of specific practices such as targeted food aid and survey interventions in relation to wider social‐political environments (Collinson et al., 2010; Manyena, 2009). These studies are complemented by works examining the unintended consequences of disaster relief written from a range of social locations and analytical perspectives (Ardalan et al., 2019, Jackson, 2019). Our research shows how early community response is of significant help to those who have been hardest hit by the lockdown and how community resilience has played an essential role in tackling disaster. Furthermore, we argue that government officials can adapt to the situation and respond promptly by (1) decentralizing recovery processes to fast‐track aid distribution, (2) empowering the local leadership, and (3) establishing stronger relationships between central and local government bodies.

Our study contributes to the literature of disaster and community resilience in three ways. *First*, the existing literature on disaster management indicates that local community members are at the frontline in preparing for and dealing with the aftermath of disasters (Aldrich, 2017; Imperiale & Vanclay, 2020b, 2021; Mayer, 2019). The literature in this area further suggests that neighbourhood‐based organizing and social networking are key and have been effective in past disasters, promoting the notion that collective local action is well‐placed to address both short‐ and long‐term recovery needs (Aldrich, 2017; Cavaye & Ross, 2019; Graham et al., 2016; Imperiale & Vanclay, 2021; Pfefferbaum et al., 2017). Such action also supports community leaders in securing government resources (Thornley et al., 2015) and cultivating social connections to bolster support (Chamlee‐Wright, 2010), while fostering a strong sense of community that facilitates a return to normal (Cheshire et al., 2015). Consistent with prior research, our findings indicate that the local neighbourhood and local leadership were at the forefront of collective activity to help those who were badly affected by the lockdown. As community members, the local politicians used their social networks, political influence, and government resources to raise money for the vulnerable, while solvent neighbours took responsibility for providing food for the vulnerable over an extended period. Hence, community resilience is achieved through community networks that connect individuals to varying degrees (Aldrich, 2017; Aldrich & Meyer, 2015; Bourdieu, 1986).

Our findings are consistent with recent observations by scholars that community development can be considered as a process of engagement, empowerment, and action that fosters community resilience, and that such community principles build specific aspects of resilience such as adaptive capacity, interactions within overall systems, and equity (Cavaye & Ross, 2019; Imperiale & Vanclay, 2021; Matarrita‐Cascante et al., 2017). However, that said, it is important to build a mechanism in which a transparent accountability of resources and funds should be ensured, as recent research indicates that empowerment of local political leaders may lead to elite capture, rent seeking, and corruption (Imperiale & Vanclay, 2020a, 2020b, 2020c). These findings are more pertinent to the context of developing countries, where corruption is high, and where lack of a transparent accountability mechanism may lead to ‘disaster capitalism’.

*Second*, previous research on community resilience shows that volunteers play a vital role in catastrophe recovery and are at the forefront of disaster recovery (Aldrich, 2019; Max, 2021; Pfefferbaum et al., 2017; Thornley et al., 2015). However, research in this area focuses on the role of volunteers in the context of natural hazard‐related disasters in which they rescue others without any obligation to the environment, contrary to the case in a pandemic. Our study adds to this stream of research and indicates how whole communities respond during a pandemic. In our study, volunteers could not operate and participate freely because of government restrictions and fear of spreading the virus. Our research findings are consistent with prior studies to the extent that the role of volunteers was key to the recovery process; however, our findings indicate that education in social distancing, use of masks and proper sanitization was a crucial part of the training process before deploying the volunteers. We found that young volunteers were key to distributing food and supplies to the vulnerable. The volunteers were recruited by community leaders to assist police and local agencies in bringing support to the poorer sectors of the communities. The role of volunteers in community rebuilding is particularly pertinent to developing countries since they are generally lacking in adequate emergency management systems as well as in trust between the police and local communities. Historically, marginalized communities in developing countries have found it hard to trust the police, whereas local volunteers can more legitimately promote effective coordination and collaboration between government authorities and hard‐hit neighbourhoods (Seebauer & Babcicky, 2018). Thus, in developing countries, legitimizing and mobilizing local volunteers could be more effective than involving traditionally suppressive authorities or ‘command and control’ systems (Imperiale & Vanclay, 2020a).

*Third*, existing research into community resilience has focused more on the efforts of governments (Pathak & Ahmad, 2018), external bodies (Islam & Walkerden, 2015) and neighbours (Aldrich & Meyer, 2015), while largely overlooking the highly significant role of religious scholars and community elders in coordinating and activating a concerted community response to crisis or disaster. Our research, therefore, adds to the corpus of community resilience literature and widens the debate by emphasizing the importance of these informal and yet strongly influential community actors. In a religion‐dominant society, the power of religious scholars and clerics to generate a strong and effective community response should not be underestimated. As highly trusted and respected members of society, they can work very much at the local level to help their communities find hope and rebuild broken lives. It must be acknowledged, though, that while clerics have huge potential to positively influence disaster outcomes at the local level, one disadvantage to this may be a tendency to propagate fundamentalist ideas at a time when individuals are most vulnerable, both physically and mentally.

Our study also has important ramifications for practice. The pandemic is still ongoing, and many countries are facing a third wave of infections, pushing the vulnerable community towards even greater social inequalities. Thus, our study findings are even more relevant to local and central governments as they attempt to stabilize and recover from the social and economic costs of lockdowns. Our findings indicate that governments need to move away from central command and control tactics and instead invest in empowering local leadership and local communities. Although our research was carried out in Phulbari, Dinajpur, Bangladesh, the findings are still generalizable to many other countries that have been severely impacted by COVID‐19 and which share similar socioeconomic structures and contextual factors.

## 6 CONCLUSION

The current COVID‐19 pandemic has been disastrous for many countries and is hugely impacting human lives and economies. As an anti‐pandemic strategy, lockdown has been a practical way of slowing the virus spread. However, developing countries are among the hardest hit by lockdown, where the strategy is producing a large‐scale human crisis of poverty and starvation that threatens the survival of the marginalized communities. The lesson we learned from Phulbari is that community resilience becomes crucial to tackle a humanitarian crisis in the making, particularly in countries with inadequate disaster management plans and limited government capacity.

The difference between previous disaster situations and the current pandemic is that relief efforts are hampered at the socio‐ecological and interactional levels by lockdowns and social distancing rules. Our research of the early community response to COVID‐19 in Phulbari explores the dynamics of community resilience in a completely unprecedented pandemic situation. In so doing, we identify the most effective components of community resilience as these dynamics are adapted and revised in the face of ever‐changing conditions. In particular, we highlight the roles of religious scholars, young volunteers and community elders in bringing about community action by examining it as both a *cultural* tool and a *social* tool to help the vulnerable people. There remains, however a clear need for further research to investigate the potential of community resilience in disaster recovery.

We make three important suggestions for future disaster preparation. *First*, there is need for vastly improved communication between local government and central government. Central government can support local regions by funding recovery programs or with the help of external donors post the COVID‐19 pandemic. Since corruption is a long‐standing issue in developing countries, there needs to be transparent transfer of relief funds with minimal bureaucracy to avoid the chances of elite capture and disaster capitalism. Governments may be able to use pre‐existing systems for this – for example, social care programs such as India's ration system and the Ehsaas program in Pakistan.

*Second*, to build back a better and more resilient community, countries need to move away from centralized civil protection and towards decentralized and socially sustainable community empowerment systems. Such systems can then organize their own effective disaster management and development interventions. They can also facilitate inclusive social learning and socially sustainable transformation towards reduced local vulnerabilities, risk impacts, and the root causes of disasters, as well as enhance disaster risk reduction and resilience at all levels of society (Imperiale & Vanclay, 2020a). Community health and wellbeing initiatives should be based on well‐designed systems that empower all members of a community, particularly the most vulnerable. Over time, these systems should help to build and strengthen community culture, thus helping to avert the rise of disaster capitalism. External actors and local communities can thereby change ‘*affected* landscapes’ into ‘landscapes of *affect*’ and preserve their community structures and resources from those who would exploit them (Imperiale & Vanclay, 2020, p 553).

*Third*, in the long term, developing countries need to re‐evaluate and prioritize the primacy of people and invest more heavily in their wellbeing and disaster management programs. The irony of developing countries is that large chunks of budget are spent on military and defense while considerably less is given to healthcare, education, and the building of institutions. For example, Pakistan spends 16% of its budget on the military and only 3% on health and education. Likewise, in India, 18% of the budget is reserved for the military, while only 5% is given to health and 6% to education (Sharif & Afshan, 2018). The effects of such a discrepancy become more pronounced in times of disaster or crisis. Henceforth, it has become increasingly obvious that developing countries need to prioritize social welfare and ensure that everyone's basic needs are met.

## Data Availability

Restricted.
